# Empowering Cancer Therapy: Comparing PEGylated and Non-PEGylated Niosomes Loaded with Curcumin and Doxorubicin on MCF-7 Cell Line

**DOI:** 10.3390/bioengineering10101159

**Published:** 2023-10-02

**Authors:** Shaghayegh Saharkhiz, Atefeh Zarepour, Ali Zarrabi

**Affiliations:** 1 Department of Biotechnology, Faculty of Biological Science and Technology, University of Isfahan, Isfahan 81746-73441, Iran; 2 Department of Biomedical Engineering, Faculty of Engineering and Natural Sciences, Istinye University, Istanbul 34396, Türkiye

**Keywords:** cancer, co-loading, drug delivery, curcumin, doxorubicin, PEGylation

## Abstract

Cancer remains an enduring challenge in modern society, prompting relentless pursuits to confront its complexities. However, resistance often emerges against conventional treatments, driven by their inherent limitations such as adverse effects and limited solubility. Herein, we spotlight a remarkable solution; a niosomal platform engineered to tandemly ferry two potent agents, doxorubicin (DOX) and curcumin (CUR). Notably, we delve into the pivotal role of PEGylation, unraveling its impact on therapeutic efficacy. These niosomes consist of Span 60, Tween 60, and cholesterol with a molar ratio of 5:2:3, which were prepared via a thin film hydration method. The physicochemical characterization of particles was performed using DLS, zeta potential measurement, SEM, and FTIR analysis. In addition, their encapsulation efficiency and release profile were determined using the HPLC method. Finally, their cytotoxicity and biocompatibility effects were checked by performing an MTT assay test on the MCF7 and L929 cell lines. The obtained results confirmed the successful fabrication of co-loaded niosomal structures with and without PEG coating. The fabricated nanoparticles had sizes in the range of 100 to 200 nm with a surface charge of about −18 mV for particles without PEG coating and −40 mV for coated particles. Notably, DOX encapsulation efficiency leaps from 20% to 62% in the transition from uncoated to coated, while CUR exhibits an impressive surge from 80% to 95%. The drug release was more controlled and slower in the coated sample. Finally, the MTT results confirmed the biocompatibility and synergistic effect of the simultaneous use of two drugs on cancer cells in the PEGylated niosomal particle. Based on the results, PEGylated niosomal particles can be considered adept vehicles for the simultaneous delivery of different chemotherapy cargoes with synergic interaction to overcome cancer.

## 1. Introduction

The goal of most cancer treatments is to limit tumor cell proliferation and prevent metastasis [1]. Nevertheless, a significant portion of these efforts have failed due to drug resistance or relapse of tumors [2]. Doxorubicin is an anthracycline drug and a common chemotherapy drug that is used clinically in many malignant cancers. It shows intercalation into DNA via disrupting the function of topoisomerase-II-mediated DNA repair and also generates free radicals that damage DNA, proteins, and the cellular membrane of the cells [3]. Despite its effectiveness in the treatment of cancer, doxorubicin hydrochloride has recently been limited in clinical use due to cardiac side effects and drug resistance [4]. On the other hand, plant-based drugs such as curcumin, by affecting various intracellular pathways, play a role in cancer treatment. To illustrate, Curcumin inhibits the expression of AP-1 (Activating protein-1), EGR-1 (early growth response-1), and NFκβ (Nuclear factor-κβ), which are involved in inflammatory pathways and cell growth, with a protective effect in the treatment and prevention of cancer [5]. In addition to its poor aqueous solubility, rapid metabolism, short half-life, and low absorption in the digestive system have hindered curcumin’s effectiveness [6].

In recent years, nanotechnology, via creating ideal carriers with biocompatibility properties, capable of carrying a high dose of medicine, and stability in blood circulation, has attracted lots of attention [7]. Niosomes are one of the members of the vesicle systems superfamily that are composed of non-ionic surfactants and cholesterol. Factors that affect the formation of niosomes are the type of surfactant, encapsulated drug, the temperature at which the lipids are hydrated, and the critical aggregation parameter [8]. The production of niosomes has been investigated first in the cosmetic industry and then with potential use in drug delivery. Although niosomes are considered to be an analog of liposomes, they have a significant advantage over liposomes in terms of cost-effectiveness, drug entrapment, and bioavailability [9]. In addition, the surfactants used in creating niosomes are more biocompatible, biodegradable, and non-immunogenic. These systems can load both hydrophilic and hydrophobic drugs, where hydrophilic drugs are placed in the central aqueous part and hydrophobic drugs are fixed between the bilayers of the niosome membrane [10].

They deliver therapeutic compounds to the intended site and reduce the side effects on normal tissues and cells [11]. To overcome the weaknesses of these carriers such as drug leakage, we can coat their surface with biocompatible polymers like Polyethylene Glycol (PEG). The PEGylation strategy, in addition to eliminating the drug leakage from the niosomal particles, promotes the biocompatibility, stability, and shelf-life of the carriers and reduces the probability of their immunogenicity [12].

The aim of this research is to fabricate a type of niosomal formulation that contains both hydrophobic and hydrophilic therapeutic compounds, simultaneously, for cancer treatment. In addition, we have compared the effectiveness of PEGylation on different features of the fabricated niosomes. To reach these aims, drug-loaded niosomal formulations were fabricated via the thin-film hydration method, and the physiochemical properties of both coated and noncoated systems, in terms of stability, drug loading, drug release, morphology, size, and surface charge, were detected and compared. Additionally, their biological activity was studied to check their cytotoxicity performance on both healthy and cancerous cell lines.

## 2. Materials and Methods

### 2.1. Materials

Sorbitan monostearate 60 (Span 60, ≥99%), polyoxyethylene-sorbitan-monostearate (Tween 60, ≥99%), cholesterol (Chol, ≥99%), Polyethylene Glycol 2000 (PEG2000), Trifluoroacetic acid (TFA), Acetonitrile, Dulbecco’s modified Eagle’s medium (DMEM) including 10% fetal bovine serum (FBS), thiazolyl blue tetrazolium bromide (MTT) powder, phosphate-buffered saline salts (PBS), and dimethylsulfoxide (DMSO) were purchased from Sigma, St. Louis, MO, USA. Isopropyl alcohol (≥99.9%), chloroform (≥99.9%), and ethanol (≥99.9%) were bought from Merck, Darmstadt, Germany. Curcumin (≥99%) and doxorubicin hydrochloride (DOX-HCl, ≥99%) were purchased from Actore Co, Karaj, Iran. In addition, ThermoFisher Scientific Inc, (Bremen, Germany) provided penicillin/streptomycin (0.5%) and trypsin/EDTA (0.5%).

### 2.2. Construction of Niosomes without Coating and with PEG Polymer Coating

For the fabrication of noisome, Span 60, Tween 60, and cholesterol with a molar ratio of about 5:2:3 along with 5 mg of curcumin were dissolved in 10 mL of chloroform and stirred for 15 min. Then, the organic phase of this solution was removed using a rotary evaporator (STEROGLASSsr1, STRIKE 202, San Martino in Campo, Perugia, Italy) at 45 °C with an approximate speed of 150 rpm to form a thin lipid film [13]. To generate multi-layered niosomes, 10 mL of pre-heated water (60 °C) containing 500 mM of ammonium sulfate salt was added to the lipid layer and stirred for 30 min to form multi-lamellar niosomes with a pH gradient, and then 10 mL of doxorubicin solution in water (with a concentration of 0.5 mg/mL) was added to the solution and stirred for 30 min. To refine the structure, the multi-layered niosomes were subjected to an hour-long sonication process using a probe sonicator (Epishear, Shinjuku-ku, Tokyo, Japan) under controlled conditions of 0 °C. The resultant entities were then subjected to purification using a 12 kDa dialysis bag (Merck, Darmstadt, Germany), culminating in the isolation of the drug-loaded niosomes. The same process was applied for the fabrication of PEG-coated niosomes using the 5:2:3:1 molar ratio of Span: Tween: cholesterol: PEG were used [14].

### 2.3. Characterization

Surface functional groups of the niosomes underwent scrutiny via infrared spectroscopic analysis (JASCO FTIR 6300, Ishikawamachi Hachioji-shi, Tokyo, Japan), encompassing the spectral range of 400–4000 cm^−1^. The size and surface charge of niosomes made with one drug and two drugs simultaneously before and after coating with polymer were investigated by dynamic light scattering (DLS) and zeta potential analysis (HORIBA, Scientific SZ-100, Minami-ku, Kyoto, Japan). The morphology of the resultant niosomes was determined by scanning electron microscopy (SEM, Leo, 1430 VP, Zeiss, Oberkochen, Baden-Württemberg, Germany).

### 2.4. Bio-Activity Assessments

#### 2.4.1. Evaluation of Encapsulation Efficiency (EE) in Niosomes with Two Drugs In Vitro

To quantify the encapsulated quantities of DOX and CUR within the niosomes, 300 µL of drug-loaded niosomal suspension was mixed with 2700 µL of 2-propanol 1% w/v solution in water, and the solution was shaken for 48 h at room temperature. Then, it was centrifuged for 10 min at 10,000 RPM and the supernatant was separated. Next, the high-performance liquid chromatography (HPLC, KNAUER AZURA^®^, Berlin, Germany) method was used to determine the exact amount of two drugs entrapped in the niosomal formulation. Briefly, 40 µL of the supernatant was added to the Eurospher II column 100-5 C18 4.6 × 250 mm over the period of 20 min and a controlled flow rate of 1 mL/min. Notably, absorbance measurements occurred at the wavelengths of 250 and 485 nm for doxorubicin, and 420 nm for curcumin. The mobile phase encompassed a balanced blend of methanol and water, infused with 0.2% trifluoroacetic acid (TFA) and acetonitrile, orchestrated with diverse molar ratios as delineated in Table 1 [15].

The encapsulation efficiency of both drugs was calculated using the following Equation (1):

Encapsulation Efficiency (%) = Mass (loaded drug)/Mass (total drug) × 100(1)

#### 2.4.2. Stability Evaluation of the Particles

After 4 months of storage of the fabricated particles at the controlled temperature of 4 °C, three analyses of DLS, SEM, and encapsulation efficiency measurement were repeated on both PEGylated and non-PEGylated co-loaded niosomes to track their changes during the storage time.

#### 2.4.3. Drug Release Determination

To elucidate the release pattern of niosomes (with/without coating), drug-loaded particles were dispersed in two different pHs of 6.6 and 7.4 PBS, which simulated the pH of cancerous and normal tissue, respectively. Suspension of drug-loaded samples was put into dialysis bags (12 kDa) and then immersed in PBS solutions (with pH 6.6 and 7.4) and then incubated for 10 days at 37 °C. At designated intervals (2, 4, 6, 12, 24, 48, 72, 96, 144, and 240 h), discreet samples were extracted from the outer solution, and the amounts of the released drug were determined via HPLC analyses using 420 nm and 480 nm wavelengths for DOX and curcumin, respectively.

#### 2.4.4. Cytotoxicity Investigations

The evaluation of cytotoxicity was undertaken employing two distinct cellular entities: the human breast cancer cell line (MCF-7) and the healthy mouse fibroblast cell line (L-929). In brief, 1 × 10^4^ cells of each cell line were implanted in each well of 96-well plates undergoing a 24 h incubation period at 37 °C with 5% CO_2_. Next, the medium of each well was replaced with 200 µL of fresh one, including various treatments with two concentrations (10 µg/mL and 80 µg/mL). Fresh media and media containing free concentrations of drugs were used as negative and positive controls, respectively. The treated cells were incubated for 48 h at 37 °C. After that, cells were washed with PBS (twice) and then incubated for 4 h with a 100 µL culture medium containing MTT solution (5 mg/mL). In the next step, the medium of each well was replaced by 100 µL DMSO and incubated for an hour in the dark, followed by reading the absorbance of the wells with an ELISA reader (Bio-Rad, Hercules, CA, USA) at 570 nm. In addition, the pivotal parameter of inhibitory concentration (IC50) values was deduced for each treatment category employing Equation (2). This value serves as a cardinal metric, elucidating the concentration of treatment requisite to curtail 50% of cell vitality.

CI = a/A + b/B(2)

This value represents doxorubicin IC50 when combined with curcumin at concentration b, and A represents dox IC50 when used alone, while B represents curcumin IC50 when used individually. The numerical value of the Combination Index (CI) stands as a pivotal indicator, deciphering the interplay between the two compounds; a value higher than 1 indicates antagonistic interaction and a value of 1 indicates additive interaction [16,17,18].

### 2.5. Statical Analysis

To evaluate the significance of quantitative data, SPSS software (version 21, parametric analysis of variance, ANOVA (Tukey)) was used. It is noticeable that the *p* value ≤ 0.05 was used for MTT experiments.

## 3. Results

### 3.1. Characterizations

#### 3.1.1. Size and Surface Charge Determining of Niosomes

The size of niosomes containing DOX or CUR (DOX loaded niosome and CUR loaded niosome), and the combination of them (Co-loaded niosome) before and after coating with PEG2000 polymers was investigated using DLS analysis (Table 2). As can be seen, the size of the obtained particles is between 150 and 280 nm, and CUR-loaded niosomes showed bigger size than DOX-loaded ones, which can be attributed to their placement between the niosomal bilayer due to their hydrophobic nature [19]. The simultaneous incorporation of two drugs does not have much effect on the particle size, whereas their coating with PEG polymer caused a slight increase in the particle size. Additionally, the consistently observed PDI value, approximating 0.3 across all samples, corroborates the uniformity prevailing within the niosomal populations across all variations. It should be noted that the results obtained from the DLS test are the hydrodynamic radius of the particles and it is expected that their actual size is smaller than the DLS results.

Additionally, Zeta potential analysis was performed to determine the surface charge of the particles before and after PEGylation. Actually, the surface charge of the particles can affect their interaction with each other (which could lead to aggregation of the particles) and also with the cellular membrane (which could affect their cellular uptake). Therefore it is important to measure the surface charge of the particles. As is shown in Table 2, in the case of samples without PEG, zeta potential was changed, whereas all of them showed negative surface charge. Indeed, in the samples that contained DOX (DOX- and Co-loaded Niosome), the samples showed more negative surface charge that could be attributed to the adsorption of ammonium derivatives (that were used during loading of DOX) on the surface of niosomes, as well as the charged DOX, colocalized in the same portion of nanocarriers; whereas, in the case of Cur-loaded niosome, just a few changes in the zeta potential were observed (from −1 in the case of bare niosome to about −4.9 in Cur-loaded sample). After PEGylation, the surface charge of the particles significantly changed to more negative amounts of approximately −40 mV, which could be due to the hydroxyl group of the PEG molecules.

#### 3.1.2. Fourier-Transform Infrared Spectroscopy Analysis

**Figure 1:**
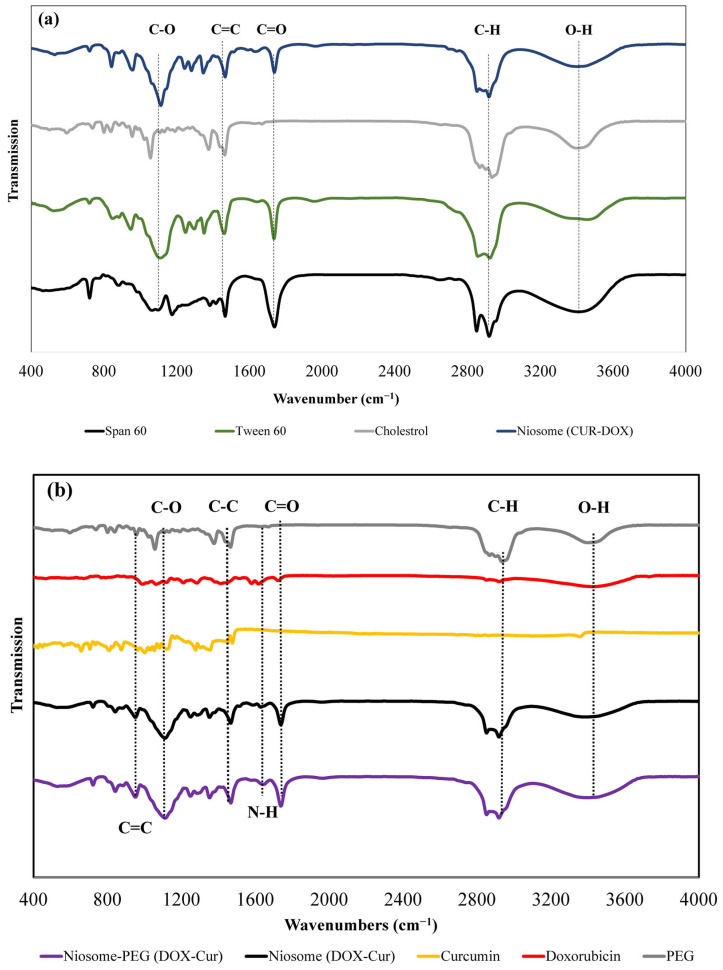
The FTIR spectrum of (**a**) empty niosomes and their structural components, and (**b**) co-loaded niosomes with and without PEG, CUR, and DOX.

To evaluate the interactions between the niosomal components, FTIR analysis was performed (Figure 1a). The obtained data demonstrated that a band was observed around 1052–1092 cm^−1^, which is attributed to the C-O vibrating bond of cyclohexane in Span 60 and cholesterol. Additionally, bands at around 1730 cm^−1^ and 2929 cm^−1^ were related to the C = O stretching vibration bond of ester groups in Span 60 or Tween 60 and the C-H bond of the carboxylic group in cholesterol, respectively [20]. In addition, another band around 3500 cm^−1^ was seen in all components that were attributed to the O-H bond of the hydroxyl group [21]. Figure 1b shows the spectrum of the DOX, CUR, and co-loaded niosomes with and without PEG coating. The results revealed that the band related to the stretching O-H group was deeper in the PEG-coated NPs in comparison to the non-coated ones, which can be related to the placement of PEG polymers on the surface of the niosomes. However, since we used the transmittance form of FTIR and most of the characteristics peaks of PEG that overlapped with the other compounds of niosome, we could not see significant differences between PEGylated and non-PEGylated niosomes. Also, two bands at 1623 cm^−1^ and 940–1100 cm^−1^ were observed in co-drug-loaded particles, which are attributed to the N- H and C = C bonds of the CUR and DOX drugs, respectively [22].

#### 3.1.3. Scanning Electron Microscopy (SEM)

**Figure 2:**
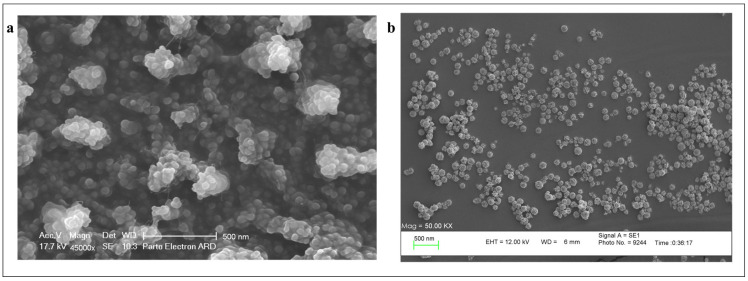
SEM images of DOX and CUR co-loaded niosome (**a**) without PEG-coating, and (**b**) with PEG-coating (Scale bar = 500 nm).

Scanning electron microscopy (SEM) analysis was performed to evaluate the morphology of DOX and CUR co-loaded niosomes with and without PEG-coating. As is shown in Figure 2, both niosomal samples have smooth surfaces and round shapes with a size of 50–200 nm. Significant aggregation was seen in the noncoated samples (Figure 2a), whereas very low aggregation was seen in the PEGylated one (Figure 2b). Also, the PEG-coated sample exhibited a more uniform and homogeneous disposition in comparison to the non-PEGylated niosomes. This observation underscores the efficacy of PEGylation in orchestrating a harmonious arrangement for the niosomal constructs that could prevent aggregation of the particles by creating distance between them.

### 3.2. Bioactivity Assessments

#### 3.2.1. Encapsulation Efficiency (EE%) Measurement

Quantitative elucidation of encapsulation efficiency (EE%) for DOX and CUR within the niosomal carriers was meticulously pursued via the HPLC method. The results of this study (Table 3) showed EE% of about 19.3% and 23.3% for DOX-loaded and co-loaded niosomes without PEG, respectively. Coating of the particles with PEG led to a significant increase in the amounts of loaded drug to 84% and 62.9% for DOX-loaded and co-loaded niosomes, respectively. We hypothesize that the reason for this noticeable enhancement of loading is the encapsulation of DOX molecules between the branches of the PEG molecules in addition to their encapsulation in the hydrophilic core of niosomes. As can be seen in the table, the amount of DOX loading in the co-loaded niosome is lower than that of DOX-loaded nanoparticles. This difference may be due to the presence of curcumin molecules between the bilayer of niosomes that prevent doxorubicin molecules from entering the niosomes based on the concentration gradient. On the other hand, the amounts of CUR EE% were not very different in the PEGylated and non-PEGylated forms and were between 80 and 95% in all types of niosomes.

#### 3.2.2. Stability Evaluation of the Particles

**Figure 3:**
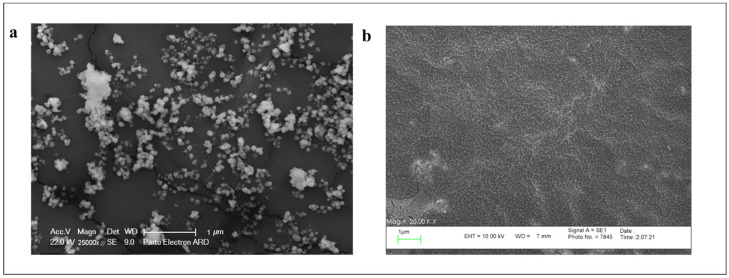
The SEM images of (**a**) non-PEGylated niosomes and (**b**) PEGylated niosomes after 4 months of storage at room temperature (Scale bar = 1 μm).

To evaluate the effect of PEGylation on the stability of niosomal particles during the time, both co-loaded particles in coated and non-coated forms were kept at 4 °C for 4 months, and then their size and morphology, as well as EE%, were measured. Based on the DLS results, the size of coated and noncoated particles changed to 306.4 nm and 499.2 nm, respectively. Furthermore, the SEM images provided significant aggregation between the particles without the PEG layer (Figure 3a), while no considerable aggregation was observed in PEGylated particles (Figure 3b). Table 4 presents the EE% of both DOX and CUR after 4 months, which shows ~46% and 13% of DOX leakage from noncoated and coated niosomes, respectively. Also, the percentage of curcumin leakage was ~25% and 20% from non-coated and coated particles, respectively. All of these data confirm better stability of coated niosomes in comparison to noncoated ones.

#### 3.2.3. Drug Release Profile of the Particles

**Figure 4:**
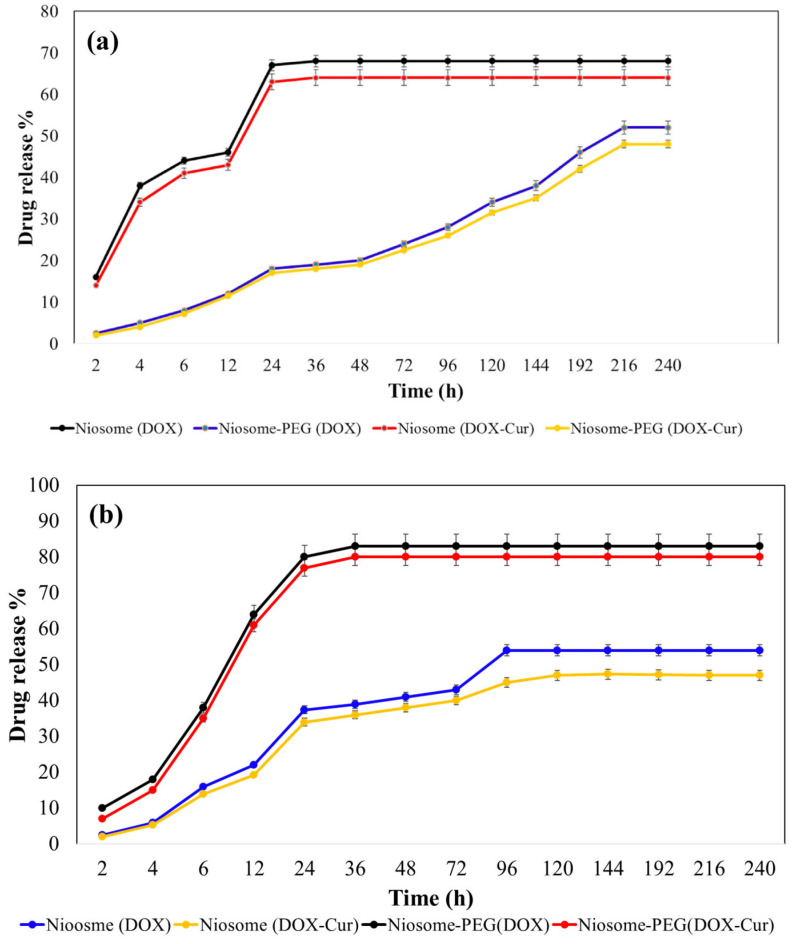

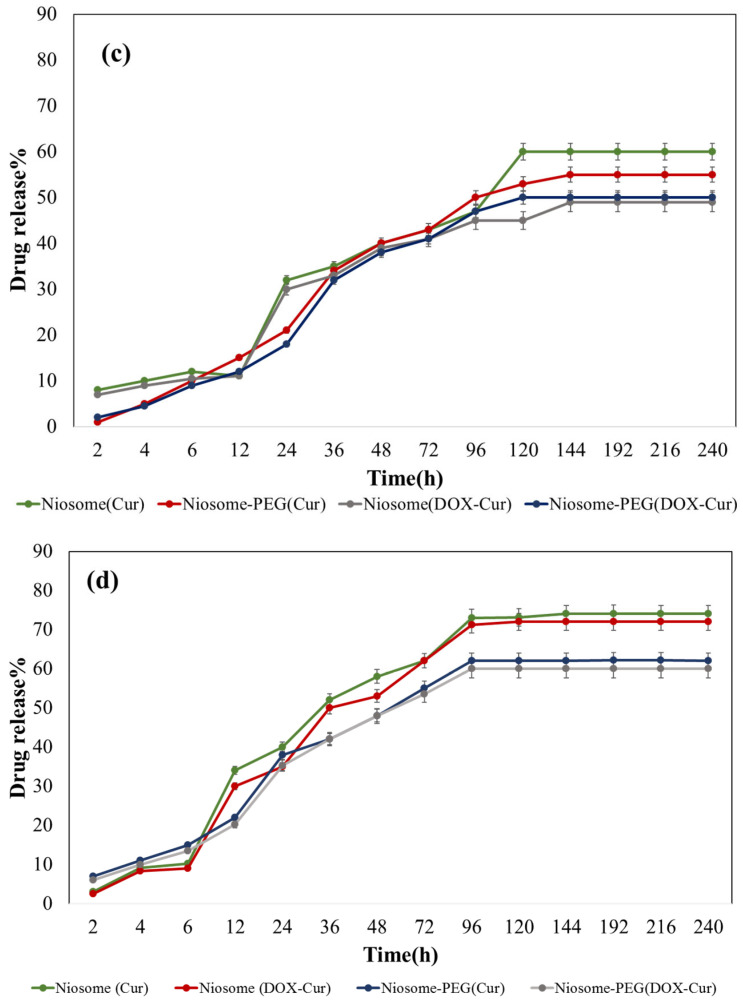
The drug release pattern of (**a**) DOX at pH of 7.4, (**b**) DOX at pH of 6.6, (**c**) CUR at pH of 7.4, and (**d**) CUR at pH of 6.6.

Figure 4 shows the release pattern of DOX and CUR from the niosomal particle in PEGylated and non-PEGylated forms in pH of 7.4 and 6.6. As it is obvious, Doxorubicin release from the niosomal particles without polyethylene glycol polymer coating had an explosive release phase in the first 6 h, during which ~55% of their encapsulated drugs released and after that, the release of the drugs became slower, and finally within 48 h, ~73% of their contents were released, and then no DOX release was observed. However, the PEGylated niosomes released only ~20% of their components in the first 6 h, which we hypothesized and is related to the entrapped DOX molecules between the PEG branches. After that, a more controlled and slower release occurred, which we assume is attributed to the encapsulated drug in the inner space of the niosomes. Finally, within 120 h, approximately 50% of the total drug was released. In the case of different pH, the release of DOX at the pH of 6.6 was more rapid in comparison to the pH of 7.4. To illustrate, in the first 4 h, 11% more DOX release was observed from DOX-Niosome at the pH of 6.6 compared to the pH of 7.4 due to the slight instability of the particles in the acidic situation [23]. Interestingly, co-loading of DOX with CUR did not affect its release behavior and only the amount of drug release was reduced slightly.

Figure 4c,d present the release profile of entrapped curcumin from both PEGylated and non-PEGylated niosomes. Based on the data, CUR release in both coated and noncoated particles occurred in a slow-release manner; however, the release occurred in a more controlled manner in the coated particles. Moreover, the PEGylated particles presented ~10% less released curcumin than non-PEGylated niosomes, and changing pH from 7.4 to 6.6 caused 12% more drug release because of the reversion of polyethylene (PE) groups of Tween60 and PEG to the hexagonal phase at the acidic environment, leading to the destabilization of the niosomes and enhancement of the drug leakage from the particles [24]. Similarly, in the case of curcumin release, no difference was seen between the curcumin release pattern in single-loading or co-loading forms.

#### 3.2.4. In Vitro MTT Assessment

**Figure 5:**
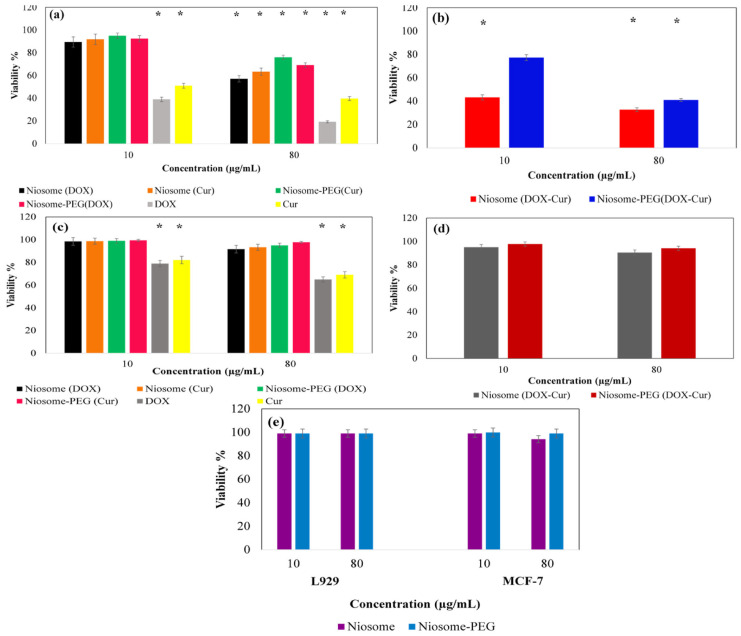
MTT assay results of different single-drug-loaded formulations of niosomes cell line (**a**), dual-drug-loaded niosomes on MCF-7 cells after 48 h of incubation (**b**), L929 cell line viability after 48 h of incubation with single-drug-loaded noisome (**c**), with dual-drug-loaded niosomal particles (**d**), and the cell viability of both MCF-7 and L929 cell lines after exposure for 48 h to empty niosomes and pure drugs (**e**) (* is related to the *p* value ≤ 0.05).

To evaluate the survival rate of the cells, MTT assay was used against MCF-7 and L929 cell lines as cancerous and normal cell lines, respectively. The results demonstrated that the drug-loaded niosomal systems reduce the growth of MCF-7 cells in a concentration-dependent manner (Figure 5). Here, 10µg/mL of free therapeutic compounds (both DOX and Cur) caused approximately 61% and 49% cell death for MCF-7 cells, respectively, whereas in the case of drug-loaded niosomes, no significant toxicity effect was seen for this concentration. This could have resulted from the differences between the amounts of the available drug in the case of free and loaded drugs. Indeed, we used the same concentration of free drug and drug-loaded samples, thus the amount of drug in the case of the free drug was more than the drug-loaded niosome. In addition, in the case of a free drug, all of the therapeutic compounds were exposed to the cells from the beginning of the test, whereas in the case of a drug-loaded sample, it took time to release the drug and so the available amounts of the drug could not show significant cytotoxicity. Increasing the concentrations of samples to about 80 µg/mL led to a significant reduction in the survival rate of cancer cells (57.15%, 69.1%, 63.36%, and 76.02% after incubation with Niosome (DOX), Niosome-PEG (DOX), Niosome (Cur), and Niosome-PEG (Cur), respectively (Figure 5a). Also, the use of co-loaded niosomes showed approximately double the toxicity on cancer cells than single drug-loaded ones (Figure 5b). In addition, a comparison between the co-loaded particles with and without PEG coating revealed that coated particles reduced cancer cell viability by ~41.30% and uncoated particles by ~32.2% at the concentration of 80 µg/mL, which confirmed the better performance of non-coated samples. This promoted cell growth inhibition of the non-coated particles in comparison to the coated ones, which could be due to the reduced cellular uptake of the PEGylated niosomes. Indeed, according to the zeta potential measurement, the PEGylated samples had a high negative charge that decreased their cellular uptake due to their electrostatic repulsion by the cellular membrane [25,26]. In addition, based on the drug release study in the current report, the PEGylation reduced the drug leakage from the particle, which can be considered as the reason for this observation. Moreover, the IC_50_ values of different types of treatments are presented in Table 5. Based on the results, the MCF-7 cells’ viability reached 50% at the concentrations of 26.4, 57.1, 36.4, and 64.6 µg/mL in Niosome (DOX), Niosome (Cur), Niosome-PEG (DOX), and Niosome-PEG (Cur), respectively. However, the half inhibitory index decreased to 15.8 and 20.7 µg/mL after the combination of two drugs in the non-coated and coated forms of niosome. In addition, the CI values of pure DOX in combination with Cur, Niosome (DOX-Cur), and PEGylated Niosome (DOX-Cur) were 0.41, 0.47, and 0.70, respectively, representing the synergic interaction between DOX and Cur in all forms, Table 6.

The effect of the synthesized nanoniosomes was also investigated on the normal cell line of L929 (Figure 5c,d). The results of this test showed that the synthesized particles did not have side effects and toxicity on healthy tissue, presenting more biocompatibility for the PEGylated particles than non-PEGylated ones (Figure 5e).

## 4. Discussion

In this study, two drugs (curcumin and doxorubicin) were simultaneously loaded in niosomal nanocarriers to improve their therapeutic effect and reduce their side effects during chemotherapy usage. Moreover, a comparison study was performed between PEGylated and nonPEGylated co-loaded niosomes. In detail, six types of niosome were prepared using Span 60/Tween 60/cholesterol with different drug contents in the form of individual drug-loaded and co-loaded with and without PEG coating on their surface. Additionally, their characteristics were evaluated using several analyses including DLS, FTIR, zeta potential, and SEM. The obtained results showed that the size of the particles was between 150 and 200 nm for the noncoated category, and betwen 200 and 300 nm for the coated ones, which can be attributed to the placement of PEG branches on the surface of the carriers [27,28]. It is noticeable that the obtained size of coated particles is an appropriate size for them to accumulate in tumor tissues via the EPR effect [29]. The second effect of PEGylation on the physiochemical features of the niosomal particles was a change of their surface charge in a way that it became more negative after PEGylation, which is attributed to the presence of hydroxyl group in the PEG polymer’s structure [30]. This negative surface charge promotes the stability of the nanocarriers over time [31]. In addition, SEM images revealed no difference in shape between coated and non-coated particles; however, the coated niosomal formulations showed more homogenous papulation than non-coated ones.

In the next step, the bioactivity of the fabricated nanoparticles was assessed. For this goal, the different types of niosomal particles were investigated in terms of EE%, stability, release patterns, and cytotoxicity. The achieved data from measuring the EE% of the loaded DOX showed that after PEGylation, its EE% increased dramatically from approximately 20% to 80%, which can be attributed to the trapping of DOX molecules between the PEG polymer branches on the surface of the particles [32]. Furthermore, the effect of PEGylation on hydrophobic CUR molecules was positive due to a change in the EE% of curcumin and increased its amount by around 15%. This observation is attributed to the PEG addition caused changes in the rigidity, hydrophobicity, chain order, and creation of space between the tails of the surfactants of the bilayer, which accordingly enhanced the EE% of CUR between the niosomal bilayer [33]. These outputs were in accordance with the study by Sacchetti et al. [34], in which PEGylated liposomes revealed more EE% than non-PEGylated ones.

Moreover, the evaluation of the stability of NPs demonstrated similar results compared to the studies by Z.liu et al. [35] and Seleci et al. [36], in which the PEGylated particles did not show much change in size and aggregation over time, whereas a significant size increase and aggregation occurred in the non-coated niosomes. The improvement of stability in the nano-niosomes due to the PEG coating could cause a reduction in drug leakage over time for both drugs, but the intensity of this effect was greater for hydrophilic drugs than the hydrophobic drugs, which may be due to their placement in the structure of the bilayer of the particles, therefore, after PEGylation, the DOX leakage rate decreased from 50% to 20%, whereas in the case of curcumin, it showed only 10% decrease in the leakage rate. Previously, an in vivo study was performed by Zhou et al. [37], who confirmed the effect of PEGylation on minimalizing the drug leakage from the particles over time. This feature could be a superiority for their storage in medical applications.

Additionally, the therapeutic effect of the particles was evaluated via MTT assay on the cancerous MCF-7 cell line and normal L929 cell line. Our outputs presented that co-loading of DOX and CUR in niosomal carriers enhanced their therapeutic potency in comparison to the single-form usage of DOX or CUR, agreeing with the study conducted by Firouzi et al. [38]. In addition, the PEGylated drug-loaded particles revealed more cytotoxicity on the cancerous MCF-7 cells than on the non-PEGylated forms while showing negligible toxicity on normal cells. This enhancement in the therapeutic effect of the NPs after PEGylation can be due to the pattern of drug release and higher EE% than noncoated ones [33]. On the other hand, more viability of the normal cells in exposure to the PEGylated forms of the particles can be attributed to the biocompatibility enhancement of the NPs after the PEGylation process. These observations were in agreement with previous work which showed the PEGylated disulfiram-loaded liposomes showed more cytotoxicity on human colorectal cancer H630 WT cells than on the non-PEGylated forms [30]. Moreover, in the previous reports, the co-encapsulation of Cisplatine and Curcumin in a chitosan-based niosome and co-loading of DOX and Quercitin in a cationic niosome were reported by M. Hemati et al., showing about 40% of cytotoxicity on the cancer cells in the combinational form. However, the combination of DOX and Cur in the current study caused a twice higher synergic effect and consequently more cytotoxicity on cancer cells than the previously mentioned works [39,40]. Overall, it can be said that the strategy of DOX and CUR co-loading in a PEG-coated niosomal particle could be considered a sufficient strategy in cancer chemotherapy.

## 5. Conclusions

The purpose of this study was to elevate the therapeutic potential of both DOX and CUR via their dual incorporation within niosomal carriers and improvement of the niosomes’ properties with the help of PEGylation on their surface. The desired NPs were fabricated successfully with a size of about 275 nm and EE% of 80% and 90% for DOX and CUR, respectively. They showed a more controllable release manner and enhanced cytotoxicity on cancer cells after PEGylation. The results confirmed that the strategy of co-loading DOX and CUR led to a synergic therapeutic effect against the cancer cells. In addition, PEGylation successfully promoted the therapeutic features, stability, and biocompatibility of the particles. So, it could be concluded that the strategy of usage of this co-loaded PEGylated niosomal particles can be considered a suitable cancer treatment.

## Figures and Tables

**Table 1:** Different molar ratios are used for HPLC analysis of DOX and CUR molecules.

Time (min)	Acetonitrile (% *v*/*v*)	Water with 0.2% TFA (% *v*/*v*)	Methanol (% *v*/*v*)
0	25	50	25
7	25	50	25
8.5	40	40	20
10	60	30	10
11.5	80	20	0
13	100	0	0
16	100	0	0
17	25	50	25
20	25	50	25

**Table 2:** DLS and zeta potential results of different nano-niosomes before and after physical coating using PEG2000 polymers.

Sample Name	Size (nm ± SD)	PDI ± SD	Surface Charge (mV ± SD)
DOX-loaded niosome	147.4 ± 1.5	0.34 ± 0.02	−19.1 ± 1.5
CUR-loaded niosome	185.9 ± 0.9	0.28 ± 0.05	−4.9 ± 0.4
Co-loaded niosome	188.9 ± 1.1	0.37 ± 0.11	−18.3 ± 1.2
DOX-loaded PEGylated niosome	196.6 ± 2.2	0.33 ± 0.09	−46.4 ± 2.3
CUR-loaded PEGylated niosome	236.8 ± 1.9	0.30 ± 0.06	−46.7 ± 1.9
Co-loaded PEGylated niosome	273.1 ± 3.2	0.39 ± 0.08	−43.2 ± 1.0

**Table 3:** Encapsulation efficiency percentages (EE%) of different types of nano-niosomes before and after physical coating using PEG2000 polymers.

Sample Name	DOX EE% ± SD	CUR EE% ± SD
DOX-loaded niosome (non-coated)	19.31 ± 1.3	-
CUR-loaded niosome (non-coated)	-	83.10 ± 3.1
DOX and CUR co-loaded niosome (non-coated)	23.31 ± 2.2	86.50 ± 4.02
DOX-loaded niosome (coated with PEG)	84.00 ± 3.1	-
CUR-loaded niosome (coated with PEG)	-	95.02 ± 2.4
DOX and CUR co-loaded niosome (coated with PEG)	62.90 ± 1.1	96.50 ± 3.7

**Table 4:** The EE% of DOX and CUR in coated and non-coated forms of co-loaded niosomal particles after 4 months.

Sample Name	EE% of DOX ± SD	EE% of CUR ± SD
DOX and CUR co-loaded niosome (non-PEGylated)	13.1 ± 1.5	61.4 ± 2.3
DOX and CUR co-loaded niosome (PEGylated)	54.3 ± 0.9	76.2 ± 3.3

**Table 5:** IC_50_ results of different treatments on the mcf-7 cell line.

Treatment Name	IC_50_ Value (µg/mL)
Niosome (DOX)	26.4 ± 2.4
Niosome (Cur)	57.1 ± 0.99
Niosome (DOX-Cur)	15.8 ± 2.1
Niosome-PEG (DOX)	36.4 ± 1.6
Niosome-PEG (Cur)	64.6 ± 1.4
Niosome-PEG (DOX-Cur)	20.7 ± 2.3

**Table 6:** CI values of different treatments on the mcf-7 cell line.

Sample	CI Value	Interaction Type
Pure DOX + Pure Cur	0.41	Synergic
Niosome (DOX-Cur)	0.45	Synergic
Niosome-PEG (DOX-Cur)	0.70	synergic

CI < 1, synergistic; CI = 1, additive; CI > 1, antagonistic.

## Data Availability

Not applicable.
